# Pars plana vitrectomy combined with pan-retinal photocoagulation, Ahmed glaucoma valve implantation, and/or phacoemulsification for complicated neovascular glaucoma treatment

**DOI:** 10.5935/0004-2749.2021-0187

**Published:** 2022-09-06

**Authors:** Derya Doganay, Selim Doganay, Cem Cankaya

**Affiliations:** 1 Eye Clinic, Çekirge State Hospital, Bursa, Turkey; 2 Department of Ophthalmology, Uludağ University School of Medicine, Bursa, Turkey; 3 Department of Ophthalmology, Inonu University School of Medicine, Malatya, Turkey

**Keywords:** Glaucoma, neovascular/complications, Vitrectomy, Glaucoma drainage implants, Phacoemulsification, Glaucoma neovascular/complicações, Vitrectomia, Implantes para drenagem de glaucoma, Facoemulsificação

## Abstract

**Purpose:**

To present long-term results of pars plana vitrectomy combined with pan-retinal endolaser photocoagulation, Ahmed glaucoma valve implantation, and/or phacoemulsification in patients with complicated neovascular glaucoma.

**Methods:**

The study comprised 15 eyes from 15 patients with neovascular glaucoma as a complication of diabetic retinopathy and owing to ischemic central retinal vein occlusion. There was a vitreous hemorrhage n all of the patients. Furthermore, 8 of the cases showed varying degrees of hyphema. All subjects received an intravitreal injection of bevacizumab three days before surgery. In 12 phakic patients, phacoemulsification, pars plana vitrectomy, and Ahmed glaucoma valve implantation were performed. Pars plana vitrectomy and Ahmed glaucoma valve implantation were performed in 3 pseudophakic patients. Perioperative and postoperative complications, intraocular pressure values, and best-corrected visual acuity scores were also recorded.

**Results:**

The mean follow-up was 24.4 ± 14.56 months. The mean preoperative intraocular pressure was 50.06 ± 7.6 mmHg. At 1 day, 7 days, and 1-, 3-, 6-, 12-month, and last visit following surgery, the mean intraocular pressure was 11.06 ± 8.22, 12.66 ± 7.27, 13.8 ± 7.73, 18.64 ± 7.05, 19.28 ± 4.61, 16.28 ± 1.68, and 16.92 ± 2.12 mmHg, respectively (p=0.001 for every follow-up visit). The mean visual acuity on the most recent appointment was 1.18 ± 0.42 logMar (p=0.001 for each subsequent visit). As postoperative early complications, varying degrees of hyphema and fibrin reactions were recorded. During follow-up, one patient developed phthisis bulbi. In 4 cases, Ahmed glaucoma valve revision surgery was required.

**Conclusions:**

In patients with complicated neovascular glaucoma, combined surgical procedures are safe, effective, and preferable both in terms of controlling high intraocular pressure and providing reasonable visual abilities.

## Abstract (pt)

**Objetivo:**

Apresentar nossos resultados de longo período de vitrectomia pars plana combinada com fotocoagulação panretiniana com endolaser, implantação da válvula Ahmed para glaucoma e/ou facoemulsificação em pacientes com glaucoma neovascular complicado.

**Métodos:**

Foram incluídos no estudo 15 olhos de 15 pacientes com glaucoma neovascular como complicação da retinopatia diabética e devido à oclusão isquêmica da veia central da retina. Todos os casos tiveram hemorragia vítrea. Além disso, 8 dos casos apresentaram diferentes graus de hifema. A injeção intravítrea de bevacizumabe foi administrada em todos os casos 3 dias antes da cirurgia. Facoemulsificação, vitrectomia pars plana e implantação da válvula Ahmed para glaucoma foram realizadas em 12 pacientes fáquicos. A vitrectomia pars plana e a implantação da válvula Ahmed para glaucoma foram realizadas em 3 pacientes pseudofáquicos. Complicações perioperatórias e pós-operatórias, valores de pressão intraocular e valores de melhor acuidade visual corrigida pré-operatório e pós-operatório foram registrados.

**Resultados:**

O acompanhamento médio foi de 24,4 ± 14,56 meses. A média da pressão intraocular pré-operatória foi de 50,06 ± 7,6 mmHg. Em 1 dia, 7 dias e 1,3,6,12 meses, e última visita após cirurgia, a média da pressão intraocular foi de 11,06 ± 8,22, 12,66 ± 7,27, 13,8 ± 7,73, 18,64 ± 7,05, 19,28 ± 4,61, 16,28 ± 1,68 e 16,92 ± 2,12 mmHg, respectivamente (p=0,001 para cada visita de acompanhamento). A média da acuidade visual na última visita foi de 1,18 ± 0,42 logMar (p=0,001 para cada visita de acompanhamento). Vários graus de reações de hifema e fibrina foram registrados como complicações precoces pós-operatórias. Phthisis bulbi foi desenvolvido em um caso durante o acompanhamento. A cirurgia de revisão da válvula Ahmed para glaucoma foi necessária em 4 casos.

**Conclusões:**

Os procedimentos cirúrgicos combinados que realizamos são seguros, eficazes e preferenciais, tanto em termos de controle da alta pressão intraocular quanto fornecimento de habilidades visuais razoáveis em pacientes com glaucoma neovascular complicado.

## INTRODUCTION

Neovascular glaucoma (NVG) is a secondary type of glaucoma characterized by neovascularization of the anterior chamber angle and/or iris surface. Intraocular pressure increases significantly, and medical therapy has only a minor effect. It leads to severe vision loss. While it was formerly known as rubeotic glaucoma, congestive glaucoma, and diabetic hemorrhagic glaucoma, Weis et al. coined the term NVG in 1963^(1)^. Ischemia produced in the retina layer is the major mechanism in NVG. Diseases such as central retinal vein occlusion, proliferative diabetic retinopathy, ocular ischemic syndrome, and intraocular tumors are the most prevalent causes of NVG^(2)^.

Medical therapy has a poor success rate. End-stage NVG treatment should be divided into three steps. The first step is to treat and/or manage the underlying cause, the second is to treat retinal ischemia, and the third is to lower the intraocular pressure. The therapy includes medical treatment, pan-retinal photocoagula-tion, cyclophotocoagulation, filtration surgeries, seton implant surgeries, and intravitreal or intracameral vascular endothelial growth factor (VEGF) inhibitors or their combinations^(3^, ^4^,^5^,^6^,^7^,^8^, ^9)^.

Conventional glaucoma surgeries have a very poor success rate in end-stage NVG due to new vascularization in the anterior chamber angle, complete closure of the anterior chamber angle as a result of fibrovascular contraction, excessive inflammation, and excessive intraocular pressure (IOP). In these cases, treating ischemia with pan-retinal photocoagulation and/or VEGF inhibitors does not usually result in IOP reduction. The anterior chamber angle was closed by fibrovascular tissue in end-stage NVG cases. When these situations are complicated by hyphema and vitreous hemorrhage, laser photocoagulation appears to be difficult. On the other hand, corneal edema and pupil dilatation problems caused by increased IOP limit efficient retinal photocoagulation even when refractive media is clear. Seton surgeries are generally preferred in the majority of cases for these reasons. Various implants are utilized as seton surgery in NVG treatment in the literature^(6^,^7^,^8)^. However, the fact that the underlying main cause, ischemia, cannot be treated permanently, results in low anatomic and functional success in the majority of cases over the medium- and long-term period.

In this study, we would like to discuss the long-term outcomes of our combined surgeries that included pha-coemulsification (with or without cataract), intraocular lens implantation, pars plana vitrectomy (PPV) with pan-retinal endolaser photocoagulation, Ahmed glaucoma valve (AGV) implantation into the ciliary sulcus (distance between the iris and intraocular lens) in phakic patients, and consisting of PPV with pan-retinal endola-ser photocoagulation, AGV implantation into the ciliary sulcus in pseudophakic patients with complicated NVG.

## METHODS

In this study, we retrospectively evaluated the medical records of 15 patients with complicated NVG who underwent combined PPV, endolaser photocoagulation, phacoemulsification, and AGV implantation surgeries as a complication of diabetic retinopathy and central retinal vein occlusion. This study was performed in accordance with the Declaration of Helsinki, and ethics approval was obtained from the Local Ethics Committee (protocol code: 2020/727)

All patients underwent detailed ophthalmic examination including slit-lamp examination, gonioscopy as much as possible, and B-scan ultrasonography. The period of follow-up was at least 1 year.

All NVG patients were diagnosed clinically. The diagnostic criteria of the disease were as follows: iris neo-vascularization accompanying hyphema or vitreous hemorrhage, increased IOP, significantly decreased visual acuity, and past primary disease including proliferative diabetic retinopathy or central retinal vein occlusion.

All patients showed high IOP levels, vitreous hemorrhage, and/or hyphema that made retinal laser photo-coagulation impossible. The visual acuity of the patients was at the level of light sensation or hand movements. As the posterior segment could not be evaluated in all cases, B-scan ultrasonography was performed. Patients with problems other than vitreous hemorrhage in the retina were not included in the study. None of the patients had undergone PPV previously. Different degrees of corneal edema were recorded in all cases owing to high IOP levels.

### Statistical analysis

The data were analyzed using the Statistical Package for Social Sciences (SPSS version 22.0). Paired Samples Statistics test was used as a parametric test statistic as p>0.05 according to the Shapiro-Wilk test statistic and the variables were in accordance with normal distribution. P<0.05 was considered to indicate statistical significance.

### Surgical technique

**Figure 1:**
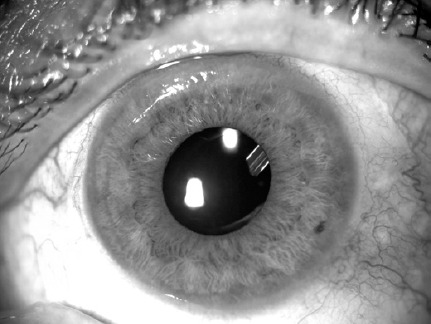
The AGV tube tip placed in the ciliary sulcus.

**Figure 2:**
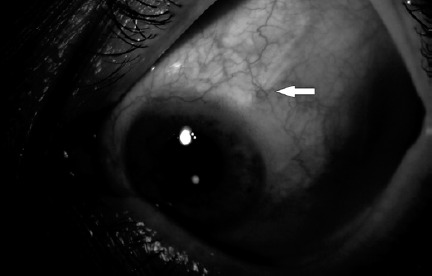
Butterfy-shaped 10-0 nylon suture (indicated with the arrow).

Intravitreal injection of bevacizumab (0.05 ml; Avastin, Roche, Switzerland) was performed in all patients 3 days before the surgery. All patients were operated on under peribulbar local anesthesia by the same surgeon (S.D). Traction sutures with 4-0 silk sutures were placed between the hours of 3 and 9. A fornix-based incision was created through the conjunctiva and tenon’s capsule from the temporal superior quadrant. Then, a 4 × 4-mm scleral flap with 50% scleral thickness was made in the same area. Mitomycin C-soaked sponges (0.2 mg/ml) were applied for 3 min to the scleral area where the AGV (FP7, New World Medical Inc., Rancho Cucamonga, CA, USA) body was placed. At the end of the application, this area was washed with 60 cc of the balance salt solution (BSS). Then, the AGV was inserted between the lateral rectus and superior rectus, and two 5-0 polyester sutures were fastened at 9 mm behind the corneal limbus. Phacoemulsification and intraocular lens implantation procedure were performed in all phakic patients, irrespective of the presence of cataracts. This step was skipped in pseudophakic cases. Then, 23-gauge PPV combined with pan-retinal endolaser photocoagulation procedure was completed. After the removal of trocars, the trocar regions were closed with 8-0 vicryl single suture. In all cases, the AGV tip was placed from the area of the scleral flap into the sulcus (in the space between the iris and the intraocular lens and the tube tip to be seen 1-1.5 mm in the pupillary cavity) (Figure 1). The tube under the scleral flap was fixed with 1 butterfly-shaped 10-0 nylon sutures without disturbing the fluid flow (Figure 2). Then, the scleral flap was closed on the tube with 10-0 nylon monofilament sutures from the corners. Conjunctiva and tenon capsules were closed with 8-0 vicryl sutures in accordance with the anatomy. In all cases, we aimed to control the postoperative inflammation by administering diluted triamcinolone to the anterior chamber. At the end of the operation, 1 ml of the subconjunctival gentamycine-onadron mixture was prepared in the lower fornix. In addition, 0.1 ml (0.2 mg/ml) mitomycin C was made to 1 ml with BSS and injected subconjunctivally in the region in accordance with the AGV body.

In cases requiring blep revision during follow-ups, the fornix-based conjunctival flap was created, and the thick fibrotic tissue formed on the seton material was removed. Then, 0.2 mg/ml mitomycin was applied to this area under the conjunctiva for 2 min and then washed with 60 cc of BSS. Next, 0.1 ml (0.2 mg/ml) mitomycin C was made to 1 ml with BSS and injected subconjunctivally in the region in accordance with the AGV body after the conjunctiva was closed.

Patients’ data, including age, gender, IOP measurements before and after the surgery, best-corrected visual acuities before and after the surgery, perioperative and postoperative complications, and postoperative medications were recorded. Regular follow-ups were conducted at 1 day, 1 week, and 1-, 3-, 6-, and 12-month after surgery.

## RESULTS

The study comprised 15 eyes (7 right, 8 left) from 15 patients with neovascular glaucoma, diagnosed as a complication of diabetic retinopathy in 11 cases and as a result of ischemic central retinal vein occlusion in 4 patients. Ten of the subjects were men and 5 women. The mean age of the patients was 62.2 ± 3.58 years. The mean follow-up was 24.4 ± 14.56 months. All cases had a vitreous hemorrhage, and 8 patients were accompanied by hyphema. Corneal edema of varying degrees was present in all cases due to increased IOP (Table 1).

**Table 1:** Characteristics of the study patients

Characteristics	
Gender	
Male	10 (66.6%)
Female	5 (33.3%)
Age (year)	62.2 ± 3.58
Eye	
Mean pre-BCVA	Light sensation or hand movements
Mean pre-IOP	50.06 ± 7.6 mmHg
PRP history	0
Pre-anti-VEGF history	0
Vitreous hemorrhage	15 (100%)
Hyphema	8 (53.3%)
Varying degrees of corneal edema	15 (100%)
Lens status	
Phakic	12 (80%)
Pseudophakic	3 (20%)
Primary disease	
DM	11 (73.3%)
CRVO	4 (26.6%)
Surgical method	
PE, PPV with endolaser, AGV implantation	12 (80%)
PPV with endolaser, AGV implantation	3 (20%)
Antiglaucomatous requirement after surgery	
Dorzolamide	4 (26.6%)
Dorzolamide and brimonidine tartrate	3 (20%)
**Total**	**15**

BCVA= best-corrected visual acuity; IOP= intraocular pressure, PRP: pan-retinal photocoagulation; DM= diabetes mellitus; CRVO= central retinal vein occlusion; PE= Phacoemülsification AGV= Ahmed glaucoma valve; PPV= Pars plana vitrectomy

**Figure 3:**
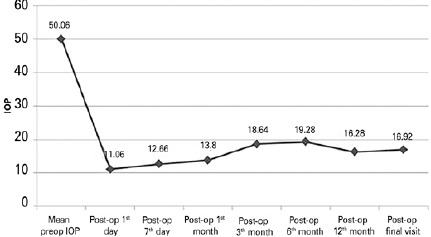
The mean preoperative and postoperative IOP alterations.

In the preoperative period, the patients’ mean IOP was 50.06 ± 7.60 mmHg. The mean IOP at 1 day, 7 days, and 1, 3, 6, 12 months, and final visit was 11.06 ± 8.22, 12.66 ± 7.27, 13.8 ± 7.73, 18.64 ± 7.05, 19.28 ± 4.61, 16.28 ± 1.68, and 16.92 ± 2.12 mmHg respectively (p=0.001 for every follow-up visit) (Table 2, Figure 3).

**Table 2:** The mean preoperative and postoperative IOP alterations

Mean pre-op IOP (mmhg)	Post-op 1^st^ day	Post-op 7^th^ day	Post-op 1^st^ month	Post-op 3^th^ month	Post-op 6^th^ month	Post-op 12^th^ month (1^st^ year)	Post-op final visit
50.06 ± 7.60	11.06 ± 8.22	12.66 ± 7.27	13.8 ± 7.73	18.64 ± 7.05	19.28 ± 4.61	16.28 ± 1.68	16.92 ± 2.12

Pre-op= preoperative; post-op= postoperative; IOP= intraocular pressure.

Before the surgery, the patients’ visual acuities were at the level of light sensation or hand movements. Except for one patient who developed phthisis bulbi, all patients’ visual acuities improved by the end of the first year after the surgery. The mean visual acuity in the last controls was 1.18 ± 0.42 logMar (p=0.001 for every follow-up visit).

During the operation, severe hemorrhage in the anterior chamber was seen in 1 case, while minimal hyphema was seen in 1 case. Minimal fibrin reaction was observed in 4 cases in the early postoperative period. In 2 cases, a serious fibrin reaction was observed, one of which was accompanied by grade 1 hyphema. Fibrin reactions and hyphema improved in all cases with medical treatment. During follow-ups, one patient developed phthisis bulbi. In this case, the patient suffered severe hyphema during the procedure, and IOP could not be managed during follow-ups, thus 3 ml of retrobulbar pure alcohol (95%) was injected into the retrobulbar cavity to reduce discomfort in the first month following surgery. This instance was also deemed inoperable. Although this case was included in our study, measurements were excluded from the quantitative evaluations. Bleb revision was necessary in 4 cases during the postoperative period due to IOP elevation that could not be controlled medically (Table 3).

**Table 3:** Postoperative complications

Hyphema	2 (13.3%)
Fibrin reaction	6 (40%)
Phtisis bulbi	1 (6.6%)
Require bleb revision	4 (26.6%)

In the last controls, IOP control was achieved in 7 cases without medication, in 4 cases with a single drug (dorzolamide), and in 3 cases with a dual drug (dorzolamide-brimonidine tartrate) (Table 1).

## DISCUSSION

Pan-retinal laser photocoagulation is the first step in the treatment of NVG. Subsequently, depending on the level of NVG, filtration surgery or seton surgery with antifibrotic drugs may be explored. In addition to argon laser photocoagulation, intravitreal bevacizumab applications are generally recommended for neovascularization regression^(10^,^11)^.

Despite extremely successful surgical treatment of glaucoma, there are still significant challenges in NVG treatment, particularly in advanced cases with vitreous hemorrhage and/or hyphema. In cases complicated with vitreous hemorrhage and hyphema, retinal ischemia cannot be treated with laser photocoagulation since the posterior segment cannot be visualized. Surgical approaches should be considered in such cases because anti-VEGF applications alone do not have a lasting effect on the underlying cause. In cases of advanced-stage NVG, complicated with hyphema and vitreous hemorrhage, aggressive surgery is occasionally required. All of our cases included in the study had these examination findings.

In complicated NVG cases, combined surgical procedures performed in the same session may cause severe bleeding in both the anterior chamber and the vitreous cavity during surgery. Preoperative anti-VEGF applications are recommended to minimize these complications, and it is stated that this application increases surgical success^(12^,^13)^. We performed a 0.05 ml intravitreal injection of bevacizumab in our cases 3 days before surgery. Except for one patient who had significant severe anterior chamber bleeding following the operation, iris neovascularization completely regressed after injection of bevacizumab before the surgery.

In our study, we observed that performing PPV, endolaser photocoagulation, phacoemulsification-in-traocular lens implantation, and AGV implantation surgeries in the same session had satisfactory results in terms of decreasing IOP and visual rehabilitation in patients with complicated NVG. We believe that the bevacizumab injection given 3 days before the surgery had a positive effect on the success of the surgery. While the mean preoperative IOP was 50.06 ± 7.6, it was detected during the final visit to be 16.92 ± 2.12. Furthermore, while the patients’ visual acuities were at the level of light sensation or hand movements before the surgery, the rates improved following the surgery, except for one patient who developed phthisis bulbi.

The purpose of PPV in complicated NVG is to eliminate intraocular hemorrhage, increase visual acuity, and enable pan-retinal photocoagulation. In cases with NVG, pan-retinal photocoagulation inhibits the formation of new vessels and promotes regression of the existing vessels in both the anterior and posterior segment by reducing the release of angiogenetic factors from the ischemic retina^(13)^. Except for the patient with phthisis bulbi, endolaser photocoagulation performed during PPV significantly prevented the formation of neovascularization in both the anterior and posterior segments in all cases.

In our investigation, independent of the existence of cataracts, phacoemulsification-intraocular lens implantation surgery was conducted in phakic patients. This process not only improved the image quality during PPV but also enabled a more effective pan-retinal photocoagulation procedure by removing the vitreous body completely.

Given that the anterior chamber angle is closed in patients with NVG due to fibrovascular tissue formed in the angle, we believe that inserting the AGV tip into the ciliary sulcus will prevent complications including long-term retraction of the tube tip, occlusion of the tip by the iris, formation of endothelial damage, and bleeding into the anterior chamber. AGV implantation into the ciliary sulcus is effective and safe^(14^, ^15^,^16)^. Except for the patient who developed phthisis bulbi, no complications linked to the tube tip were seen during follow-up and after surgery, and the tube remained stable in the ciliary sulcus. It has been revealed that inserting the tube tip into the anterior chamber might cause endothelial decompensation and graft failure in keratoplasty patients^(17^, ^18^,^19^,^20)^. According to Bayer et al., in a comparative analysis of the patients who underwent AGV implantation into the anterior chamber or ciliary sulcus, the tube tip inserted into the ciliary sulcus provides better IOP control in the medium- and long-term period^(21)^. In all of our instances, we additionally inserted an AGV tube tip into the ciliary sulcus. Similarly, to the trials, we believe that implantation of an AGV tube into the ciliary sulcus is safe and successful in terms of providing improved IOP control and fewer complications in the long run.

Encapsulation in the region where the valve body is inserted, followed by a rise in IOP, is one of the most important reasons for failure after AGV implantation surgery. To boost the chances of success, we applied 0.2 mg/ml mitomycin C for 3 minutes to the scleral surface area where the AGV body will be inserted. Furthermore, unlike routine applications, 0.2 mg/ml of mitomycin diluted to 0.1 ml with BSS was performed subconjunctivally to the area where AGV was inserted at the end of surgery. Regardless of these practices, bleb revision was necessary in 4 cases during follow-ups. According to one study, applying antifibrotic agents to the surface on which the AGV body would be placed increases the success rates when compared to the standard applications^(22)^. Another study suggested that intraoperative and postoperative mitomycin C applications increase AGV success rates and have a positive effect on the hypertensive phase^(23)^.

In NVG cases, combined surgeries might result in serious inflammatory reactions and severe bleeding into the anterior chamber and vitreous cavity. As a result, all cases received intracameral diluted triamcinolone acetonide at the end of the surgery to control the postoperative inflammation. In two of our cases, bleeding occurred during surgery, which was serious in one case, resulting in total hyphema and vitreous hemorrhage in the postoperative period. Because the IOP, in this case, could not be controlled after the surgery and the patient had serious pain, 95% retrobulbar alcohol injection was administered in the 1st month postoperatively. The case was considered inoperable. Phthisis bulbi developed as a result of this case’s follow-up. The patient who experienced grade 1 hyphema following the surgery improved with postoperative medical care. Postoperatively, minimal fibrin formation was observed in 4 cases and severe fibrin formation was observed in 2 cases. With medical therapy, these patients healed entirely.

We believe that the surgical approach we performed in complicated NVG patients with excessively high IOP, hyphema, and vitreous hemorrhage is also functionally and anatomically safe and successful. Furthermore, as in our cases, we believe that applying mitomycin C to the scleral surface where the AGV is placed, as well as applying mitomycin C subconjunctivally at the end of the surgery, may be useful in the long-term surgical success. Prospective studies with long-term series are needed to confirm and support our results and views.
